# Anti-Inflammatory Activity of *Boswellia serrata* Extracts: An *In Vitro* Study on Porcine Aortic Endothelial Cells

**DOI:** 10.1155/2018/2504305

**Published:** 2018-06-25

**Authors:** Martina Bertocchi, Gloria Isani, Federica Medici, Giulia Andreani, Irvin Tubon Usca, Paola Roncada, Monica Forni, Chiara Bernardini

**Affiliations:** Department of Veterinary Medical Sciences-DIMEVET, University of Bologna, Ozzano Emilia, Bologna 40064, Italy

## Abstract

This study is aimed at investigating the cytotoxicity, anti-inflammatory, and angiogenic activities of two *Boswellia serrata* extracts on primary culture of porcine aortic endothelial cells (pAECs). Chemical characterization of a dry extract (extract A) and a hydroenzymatic extract (extract G) of *B. serrata* was performed by HPLC using pure boswellic acids (BAs) as standard. In cultured pAECs, extract G improved cell viability, following LPS challenge, in a dose-dependent manner and did not show any toxic effect. On the other hand, extract A was toxic at higher doses and restored pAEC viability after LPS challenge only at lower doses. Pure BAs, used at the same concentrations as those determined in the phytoextracts, did not contrast LPS-induced cytotoxicity. Extract A showed proangiogenic properties at the lowest dose, and the same result was observed using pure AKBA at the corresponding concentration, whereas extract G did not show any effect on the migration capacity of endothelial cells. In conclusion, an anti-inflammatory activity of *B. serrata* extracts on endothelial cells was reported, though cytotoxicity or proliferative stimulation can occur instead of a protective effect, depending on the dose and the formulation.

## 1. Introduction

The endothelium, uniquely positioned at the interface between the vascular wall and the blood, regulates multiple functions such as maintenance of normal vascular tone, modulation of coagulation, and immune responses [1]. It is widely demonstrated that the exposure of endothelial cells to proinflammatory stressors results in the production of molecules correlated with a proadhesive, prothrombotic, and proinflammatory phenotype that contributes to vascular disorders [2, 3], including cardiovascular diseases (CVDs).

Since ancient times, the extracts from the oleo-gum resin of *Boswellia serrata* Roxb. ex Colebr. (family *Burseraceae*), also identified as Indian frankincense or Salai Guggal, have been used in traditional Ayurvedic medicine for the treatment of inflammatory diseases, including osteoarthritis and chronic bowel diseases [4–8].

The oleo-gum resin, obtained by incision of the bark, is composed by essential oil (5–9%), mucopolysaccarides (21–22%), and pure resin (65–85%), containing tetracyclic and pentacyclic triterpene acids, of which boswellic acids (BAs) are the most important bioactive molecules [4, 9, 10]. In particular, 11-keto-*β*-boswellic acid (KBA) and 3-O-acetyl-11-keto-*β*-boswellic acid (AKBA) were proposed to act as inhibitors of 5-lipoxygenase (5-LO) [11, 12]. Recently, other components of the phytocomplex, such as *β*-boswellic acid (*β*BA), have been suggested as anti-inflammatory molecules, acting through inhibition of serine protease cathepsin G (catG) and microsomal prostaglandin E synthase (mPGES) [9].

Differences in the relative amount of BAs and other components of the phytocomplex are related to the existence of different species of the genus *Boswellia*, to environmental conditions (e.g., soil composition, season, and air humidity), and to the extraction procedure [13] leading to herbal products of different composition and quality. In a previous study, seven *B. serrata* extracts were compared for their AKBA content and antioxidant power, highlighting wide variations [14]. In particular, one of the extracts obtained by bioliquefaction based on enzyme biocatalysis (hydroenzymatic extract) [15] showed interesting peculiarities. A lower content of AKBA and antioxidant power but higher activity in ex vivo tests on peripheral blood mononuclear cells (PBMCs) was determined in comparison with the dry extract [14]. In recent years, attention has also been focused on the role of other BAs, namely, KBA and *β*BA [16, 17], suggesting a possible pharmacological activity also for these BAs. Preliminary data showed wide variability in the concentration of BAs in different extracts [18]; therefore, the present research is aimed at deepening the chemical characterization of the two extracts previously studied, focusing on HPLC quantification of KBA and *β*BA. The effect of different formulations will be evaluated in comparison with the individual pure BAs in an interesting *in vitro* model: primary culture of porcine aortic endothelial cells (pAECs). With pig as an excellent model for translational medicine in the cardiovascular field [19, 20], we have previously isolated and cultured endothelial cells from thoracic aortas [21]. These primary cultures maintain a stable phenotype, and they prove to be an excellent model of study for the vascular response to different stressors [22, 23]. Therefore, pAECs were chosen as an ideal *in vitro* model to study the anti-inflammatory and angiogenic properties of the two *B. serrata* extracts in comparison with pure AKBA, KBA, and *β*BA, either individually or mixed together.

## 2. Materials and Methods

### 2.1. Chemicals and Reagents

Human endothelial SFM medium, heat-inactivated fetal bovine serum (FBS), antibiotic-antimycotic, and Dulbecco's phosphate-buffered saline (DPBS) were purchased from Gibco-Life technologies (Carlsbad CA, USA). Dimethyl sulfoxide (DMSO), trypsin EDTA solution, lipopolysaccharide (LPS) (*E. coli* 055: B5), glycerol, methanol, phosphoric acid, acetonitrile, and AKBA (batch number BCBN2928V and CAS number 67416-61-9) were purchased from Sigma-Aldrich Co. (St Louis, MO, USA). KBA and *β*BA (batch numbers 15020106 and 15010405 and CAS numbers 17019-92-0 and 631-69-6, resp.) were obtained from PhytoPlan (Heidelberg, Germany). Six out of seven samples (extracts A–F) are dry extracts of *B. serrata* oleo-gum resin. The powder is insoluble in water but soluble in methanol and dimethyl sulfoxide (DMSO). Extract G is an aqueous extract obtained by a process of bioliquefaction based on enzyme biocatalysis [15]. Briefly, the gum resin from *B. serrata* was suspended in water (1 : 10 *w*/*v*) and subjected to enzymatic digestion by xylanase, *α*-amylase, and glucosidase for 24 hours. One ml of hydroenzymatic extract is obtained from 145 mg of *B. serrata* resin (145 mg resin/ml).

### 2.2. Qualitative and Quantitative Characterization of *B. serrata* Extracts

Qualitative and quantitative analyses of *B. serrata* extracts were performed by a reversed-phase high-performance liquid chromatography (HPLC) method using the HPLC system (Beckman Coulter, Brea, CA, USA), comprising a 116 pump, a 507 automatic autosampler, a UV-Diode Array 168 detector, and integration software 32 Karat as reported by Beghelli et al. [14]. Seven samples (A–G) were analyzed for KBA and *β*BA concentrations and were prepared by dissolving extracts in methanol. KBA and *β*BA standard stock solutions were prepared by dissolving 5 mg of analytical standard in methanol (5 mL). The calibration curves were obtained by analyzing six serial dilutions (50 ppm, 25 ppm, 10 ppm, 5 ppm, 2.5 ppm, and 1 ppm) of the stock solution and by plotting the peak area measured at 260 nm against KBA concentrations and at 210 nm against *β*BA concentrations. The following equations of the curves were obtained:

(1) $$\begin{array}{c} KBA=77361x+44918,r^{2}=0.999,\\ \beta BA=26532x+721.54,r^{2}=0.999. \end{array}$$

The KBA and *β*BA peaks in the samples were identified on the basis of the retention time on the chromatogram at 260 nm and 210 nm, respectively. All measurements were performed in triplicate and data were reported as mean ± SD.

### 2.3. Cell Culture and Treatment

Porcine aortic endothelial cells (pAECs) were isolated and maintained as previously described by Bernardini et al. [21]. All experiments were performed with cells from the third to the eighth passage. The first seeding after thawing was always performed in T-25 tissue culture flasks (3 × 10^5^ cells/flask) (T-25, BD Falcon, Franklin Lakes, NJ, USA), and successive experiments were conducted in 24-well plates (scratch test) or 96-well plates (cell viability) with confluent cultures. Cells were cultured in human endothelial SFM medium, added with FBS (5%) and antimicrobial/antimycotic solution (1x) in a 5% CO_2_ atmosphere at 38.5°C. Extract A was dissolved in DMSO at 10 mg dry extract/ml (stock solution) and then diluted in culture medium to obtain four doses containing 0.1, 1, 10, and 100 *μ*g of dry extract/ml, respectively. Extract G, which is an aqueous solution, was directly diluted in culture medium to obtain four doses referring to 2.4, 24, 240, and 2400 *μ*g of resin/ml. These doses were chosen and normalized on the basis of AKBA concentration in extracts as reported in [14]: for both extracts, the lowest dose contained 3.8 ng/ml of AKBA and the highest dose contained 3.8 *μ*g/ml of AKBA.

Pure analytical grade BAs (KBA, AKBA, and *β*BA) were dissolved in methanol (stock solution 1 mg/ml) and then in culture medium to obtain the required concentrations. Two doses were chosen: *low*, corresponding to 3.8 ng/ml AKBA, 3 ng/ml KBA, and 8 ng/ml *β*BA, and *high*, corresponding to 380 ng/ml AKBA, 300 ng/ml KBA, and 800 ng/ml *β*BA. For each treatment, the same concentration of the specific vehicle was used as control.

### 2.4. Effect of *B. serrata* Extracts on pAEC Viability

pAECs were seeded in a 96-well plate (6 × 10^3^ cells/well) and exposed to four increasing doses of *B. serrata* extracts for 24 h. Cell viability was measured using tetrazolium salt (MTT assay). The formazan absorbance was measured at a wavelength of 570 nm, using Infinite® F50/Robotic absorbance microplate readers from TECAN (Life Sciences). The background absorbance of multiwell plates at 690 nm was also measured and subtracted from the 570 nm measurements.

### 2.5. Effect of *B. serrata* Extracts on LPS-Induced pAEC Death

pAECs seeded in a 96-well plate (6 × 10^3^ cells/cm^2^) were exposed to lipopolysaccharide (LPS) (25 *μ*g/ml) for 24 h either in the presence or in the absence of extracts A and G or pure BAs at the concentrations reported above. Cell viability was evaluated by MTT assay.

### 2.6. Effect of *B. serrata* Extracts on pAEC Migration Capacity

pAECs were seeded in a 24-well plate (4 × 10^4^ cells/well). When cells reached confluence, a wound was induced scratching the surface by a pipette tip, then the detached cells were removed by washing with DPBS. Complete medium containing low and high doses of extract A (0.1 *μ*g dry extract/ml and 10 *μ*g dry extract/ml) and extract G (2.4 *μ*g resin/ml and 240 *μ*g resin/ml) and pure BAs at low (3 ng/ml KBA, 3.8 ng/ml AKBA, and 8 ng/ml *β*BA) and high (300 ng/ml KBA, 380 ng/ml AKBA, and 800 ng/ml *β*BA) concentrations were added. Microscopic phase-contrast pictures and three measurements of the damaged areas were taken immediately after the scratches (T0) and after 6 h (T1) and 24 h (T2). Images were acquired using a Nikon epifluorescence microscope equipped with digital camera (Nikon, Yokohama, Japan).

### 2.7. Statistical Analysis

Each treatment was replicated three times (migration capacity) or eight times (cell viability and LPS challenge). Data were analyzed with a one-way analysis of variance (ANOVA) followed by the Tukey post hoc comparison test or Student's *t*-test. Differences of at least *p* < 0.05 were considered significant. Statistical analysis was carried out using R software (http://www.R-project.org).

## 3. Results

### 3.1. KBA and *β*BA Quantification by HPLC-DAD Analysis

**Figure 1:**
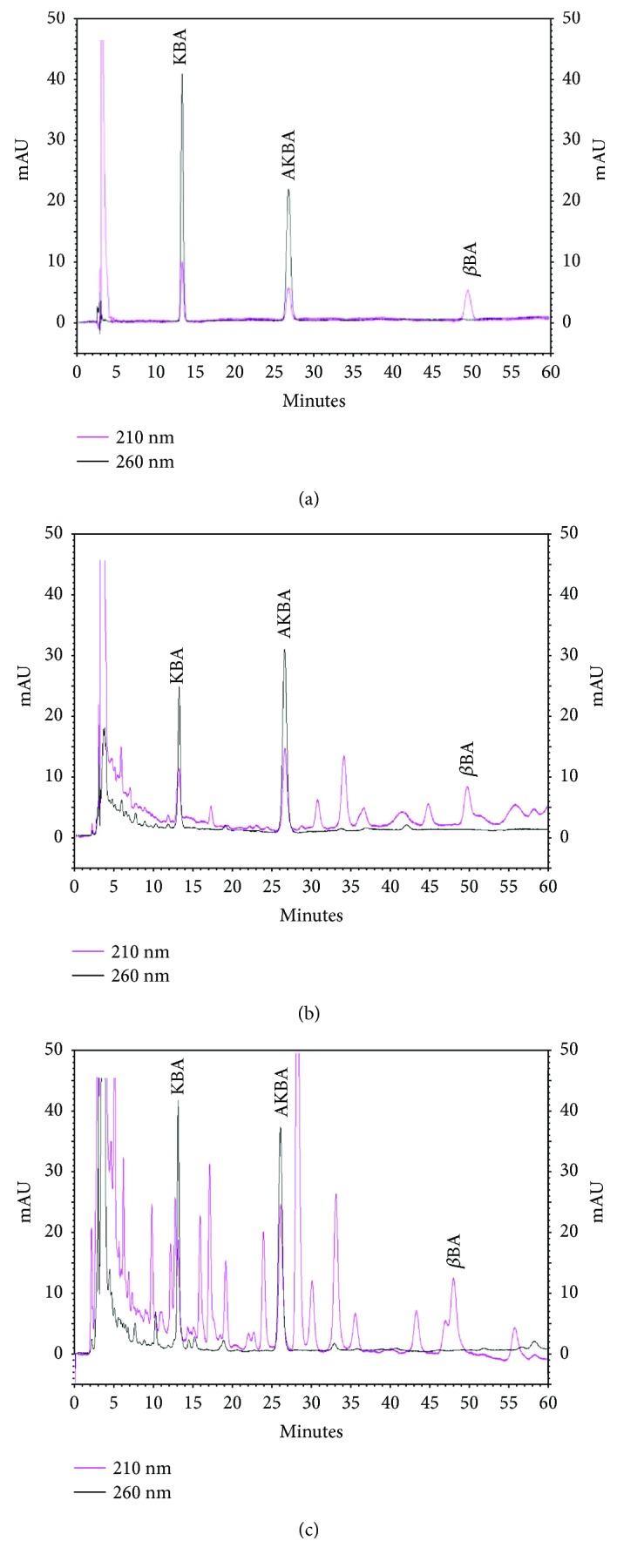
Representative chromatograms of pure analytical grade BAs (KBA, AKBA, and *β*BA) (25 ppm each) (a), extract A (b), and extract G (c) at 210 (pink chromatogram) and 260 nm (black chromatogram).

Representative chromatograms of KBA, AKBA, and *β*BA analytical standards as well as extracts A and G analyzed at 210 and 260 nm are reported in Figure 1.

Both extracts presented two major peaks at 260 nm: the first one, at Rt of 13.2 min, identified as KBA by the use of the analytical standard, and the second one, at Rt of 26 min, previously identified as AKBA. Other components of the *B. serrata* phytocomplex were only visualized at 210 nm, and the peak at Rt of 49 min was identified as *β*BA by the use of the analytical standard. KBA, AKBA, and *β*BA concentrations, calculated based on the peak area and the calibration curve, are shown in Table 1.

Quantitative and qualitative differences were present. The concentrations of BAs in extract G were two orders of magnitude lower than in extract A, and the chromatogram of extract G was characterized by a major number of peaks resolved at 210 nm. Data on KBA and *β*BA concentrations in other additional five dry extracts (B–F) are reported in Table S1 in the Supplementary Material.

### 3.2. Effect of *B. serrata* Extracts on pAEC Viability

**Figure 2:**
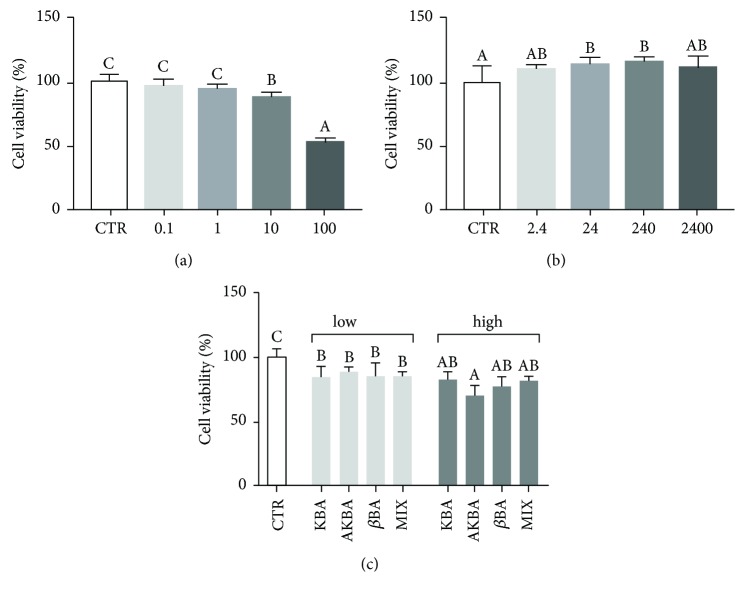
Effect of increasing doses of *B. serrata* extract A (0.1, 1, 10, and 100 *μ*g dry extract/ml) (a), extract G (2.4, 24, 240, and 2400 *μ*g resin/ml) (b), and pure BAs (*low*, corresponding to 3.8 ng/ml AKBA, 3 ng/ml KBA, and 8 ng/ml *β*BA, and *high*, corresponding to 380 ng/ml AKBA, 300 ng/ml KBA, and 800 ng/ml *β*BA) (c) on pAECs. Cell viability was measured by MTT assay. Data are reported as mean ± SD of 8 independent replicates. Different letters above the bars indicate significant differences (*p* < 0.05 ANOVA post hoc Tukey's test).

Extract A was cytotoxic at higher concentrations, resulting in a reduction in cell viability of 12 and 47%, respectively, while lower concentrations did not affect cell viability (Figure 2(a)). Extract G did not show any toxic effect on pAECs (Figure 2(b)). In the presence of pure BAs, a significant (*p* < 0.05) cytotoxic effect was detected at the concentrations studied (Figure 2(c)). Only AKBA presented a dose-dependent effect.

### 3.3. Effect of *B. serrata* Extracts on LPS-Induced pAEC Death

**Figure 3:**
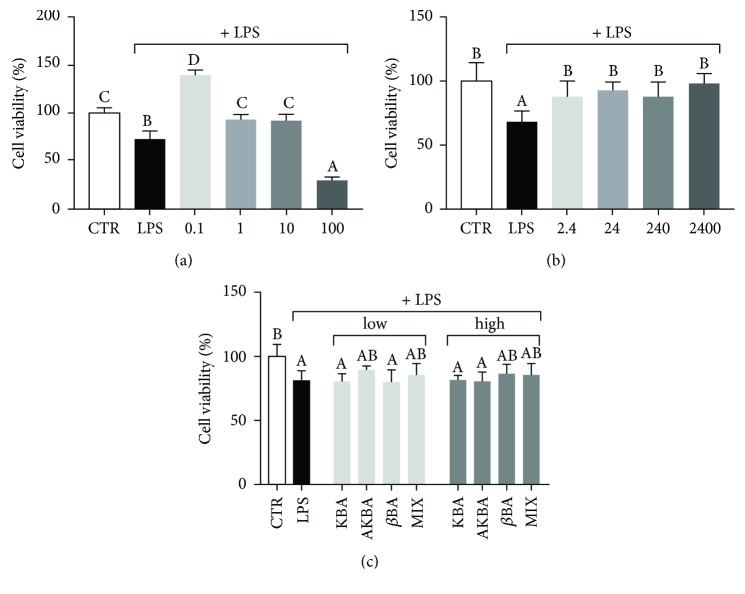
Effect of increasing doses of *B. serrata* extract A (0.1, 1, 10, and 100 *μ*g dry extract/ml) (a), extract G (2.4, 24, 240, and 2400 *μ*g resin/ml) (b), and pure BAs (*low*, corresponding to 3.8 ng/ml AKBA, 3 ng/ml KBA, and 8 ng/ml *β*BA, and *high*, corresponding to 380 ng/ml AKBA, 300 ng/ml KBA, and 800 ng/ml *β*BA) (c) on pAEC viability, in the presence of LPS (25 *μ*g/ml), measured by MTT assay. Data are reported as mean ± SD of 8 independent replicates. Different letters above the bars indicate significant differences (*p* < 0.05 ANOVA post hoc Tukey's test).

LPS challenge determined a significant 30% reduction of cell viability. Extract A significantly (*p* < 0.05) reduced the cytotoxicity induced by LPS at the lower concentrations (Figure 3(a)). The highest concentration elicited a significant exacerbation of LPS cytotoxicity resulting in 70% reduction of cell viability, while the lowest concentration showed a significant proliferative effect, resulting in a 40% increase in cell viability. Extract G significantly (*p* < 0.05) restored pAEC viability after LPS treatment at all the concentrations analyzed (Figure 3(b)), without a dose-dependent effect. None of pure BAs, individually or mixed together, was able to contrast LPS cytotoxicity (Figure 3(c)).

### 3.4. Effect of *B. serrata* Extracts on pAEC Migration Capacity

**Figure 4:**
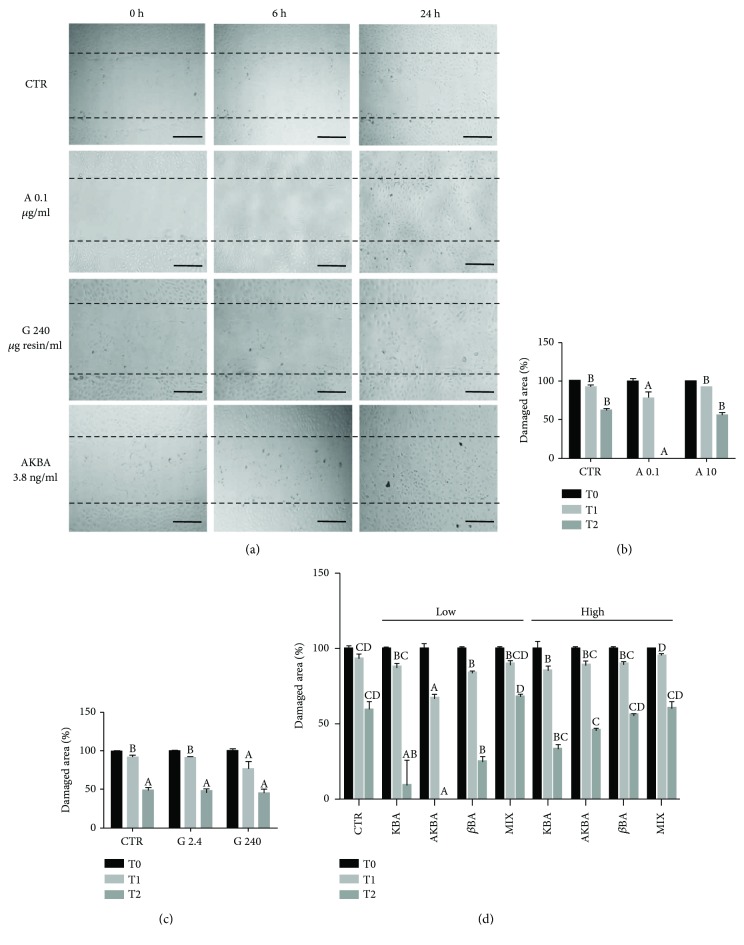
Effect of *B. serrata* extracts on pAEC migration capacity. Cells were scratch wounded and then treated with extracts A and G and pure BAs. Photographs were recorded at 0 h (T0), 6 h (T1), and 24 h (T2) after scratching. (a) Representative microscopic phase-contrast pictures showing the size of the scratch wound in different treatment groups compared with control. Scale bar, 200 *μ*m. The extent of the damaged area (%) is reported for treatment with extract A (0.1 and 10 *μ*g dry extract/ml) (b), extract G (2.4 and 240 *μ*g resin/ml) (c), and pure BAs (*low*, corresponding to 3 ng/ml KBA, 3.8 ng/ml AKBA, and 8 ng/ml *β*BA, and *high*, corresponding to 300 ng/ml KBA, 380 ng/ml AKBA, and 800 ng/ml *β*BA) (d). Data are reported as mean of 3 replicates ± SD. Inside each experimental time (T1 and T2), different letters above the bars indicate significant differences among treatments (*p* < 0.05, ANOVA post hoc Tukey's test).

Extract A reduced the damaged area at T1 (6 h) and restored completely the monolayer at T2 (24 h) at the lower concentration, while at 10 *μ*g dry extract/ml no significant effect on cell proliferation was measured (Figure 4(a)). The incubation with extract G did not determine the recovery of the damage (Figure 4(b)). Pure BAs showed a significant wound-healing effect at the end of the incubation at the lower concentration (Figure 4(c)). In particular, AKBA at 3.8 ng/ml completely restored the monolayer.

## 4. Discussion

The gum resin obtained from *B. serrata*, used in Ayurvedic medicine for the treatment of a variety of diseases, is considered a promising natural source of anti-inflammatory molecules, in particular BAs [4, 9].

The quantification of these active molecules is a prerequisite for testing any biological effect of a phytoextract from *B. serrata*. Therefore, the first aim of this study was to better characterize the BA profile through the quantification of KBA and *β*BA in addition to AKBA. The concentrations of BAs determined in extract A are in the range of those reported by other authors [24–26]. AKBA and KBA are used as markers to ensure the quality of *B. serrata* dry extracts, but their concentrations show wide variability in commercial products, which in general claim 65% of BAs. In general, BAs represent only a percentage of total organic acids, whose concentrations are determined by unspecific titration methods and, as a consequence, the claimed content of 65% BAs is absolutely unrealistic as recently pointed out also by other authors [24, 25]. Very low percentages of KBA and *β*BA were found in extract G compared to extract A. This aqueous extract was also characterized by low AKBA and low polyphenol concentrations [14], confirming again the importance of the extraction procedure on the phytocomplex composition.

To evaluate the possible biological effects of these different formulations, extracts A and G, normalized on the basis of AKBA content, were used for *in vitro* analyses to assess cytotoxicity, anti-inflammatory activity, and angiogenic properties in comparison with pure BAs. Cytotoxic effects of *B. serrata* dry extracts and BAs were reported in several studies in different cancer cell lines, such as leukemia cells, prostate cancer cells, and gastrointestinal cancer cells [7, 27–30]. As regards the biochemical mechanism of cell death, Liu et al. [31] reported that BAs are able to induce apoptosis in Hep-G2 cells through the activation of caspase-8, while Bhushan et al. [32] found that a triterpendiol derived from BAs induced apoptosis in HL-60 cells through the activation of Bcl-2 and caspase-3.

The anti-inflammatory activity of *Boswellia* extracts was demonstrated in microvascular endothelial cells by preventing TNF*α*-induced expression and activity of MMP-3, MMP-10, and MMP-12 [33]. Moreover, previous studies have shown that *B. serrata* extracts and BAs antagonize the inflammatory effect of LPS in human and mouse macrophages, monocytes, and PBMCs [34–36]. Our results demonstrated for the first time the protective effect of *B. serrata* extracts against LPS inflammatory stimulus in endothelial cells. In particular, extract G was the most effective, restoring completely cell viability at all the doses studied without any cytotoxicity. On the contrary, increasing concentrations of extract A lead to opposite results ranging from hyperproliferative effect (the lowest dose) to cytotoxic effect (the highest dose). Interestingly, in our model the use of pure KBA, *β*BA, and AKBA, either individually or mixed together, failed to protect endothelial cells from LPS toxicity and are only partially in accord with data reported by Henkel et al. [35]. In a cell-free assay, those authors suggested a direct molecular interaction between LPS and BAs lacking the keto moiety, in particular *β*BA, underlying the anti-inflammatory effect of *Boswellia* extracts.

Our results support the hypothesis that the anti-inflammatory effect of *Boswellia* extracts is not strictly dependent on the presence of the most studied BAs, but it can be related to other bioactive molecules. Other triterpenes, as incensole, could be considered interesting candidates for the pharmacological properties of frankincense, accordingly to suggestions previously reported by other authors [9, 37, 38]. Beyond these bioactive terpenes, the gum resin does contain polysaccharides. These molecules are likely to be minor components in dry extract A, whereas they can be more concentrated in extract G, due to the different polarity of the extraction medium. A water-soluble fraction extracted from the gum resin of *B. serrata* containing galactose, arabinose, and D-glucuronic acid was suggested to act as a potent enhancer of humoral and cell-mediated immune response [39], while the potential anti-inflammatory activity of these polysaccharides has not yet been explored. We cannot exclude that the polysaccharide fraction present in extract G can develop additional modulatory effects on pAECs.

The migration ability of endothelial cells is critical in the physiological and pathological angiogenesis [40]. Our results obtained with an *in vitro* model of physiological angiogenesis showed proangiogenic activity of extract A at the lowest concentration, in agreement with a proliferative effect of the same dose recorded in LPS challenge. In addition, incubation with pure AKBA at the same concentration as that measured in extract A determined the same proangiogenic effect, indicating a possible involvement of this BA in promoting angiogenesis. In contrast, incubation in the presence of extract G containing the same concentrations of AKBA did not show any effect on endothelial cell migration capacity, indicating one more time the existence of complex molecular interactions, which can modify the biological effect of the phytoextract. Contrasting results are also reported in literature. Lulli et al. [41] observed that AKBA reduced proliferation, migration, and tube formation in human retinal microvascular endothelial cells (HRMECs) stimulated with exogenous vascular endothelial growth factor (VEGF). On the other hand, Wang et al. [17] reported that *β*-BA can attenuate endothelial cell injury in a blood stasis model and protect human umbilical vein endothelial cells (HUEVCs) against cell death induced by oxygen and glucose deprivation. Different regulation pathways could be involved in the repairing activity of *Boswellia* extracts, and further investigations will be necessary to explain why different formulations determine different effects on endothelial cells pathophysiology.

How extracts of *B. serrata* gum resin should modulate the cardiovascular system has been scarcely investigated, so far. Kokkiripati et al. [42] reported that antioxidant and antithrombotic activities of extracts from *B. serrata* gum resin determined the inhibition of human monocytic cell activation and platelet aggregation. However, recently Siemoneit et al. [43] pointed out the complex agonizing and antagonizing effects of BAs on human platelet aggregation and prompted for careful evaluation of *B. serrata* extract safety in cardiovascular disease-risk patients.

In conclusion, our results demonstrate that different formulations (e.g., dry and hydroenzymatic extracts) obtained from the same botanical species show significantly different biological effects on endothelial cells. The anti-inflammatory activity of *B. serrata* extracts on endothelial cells suggests a potential pharmaceutical application for cardiovascular health, though cytotoxicity or proliferative stimulation can occur instead of a protective effect, depending on the dose and the formulation. This aspect should be carefully considered when these herbal products are used in human and animal phytotherapy.

## Figures and Tables

**Table 1:** KBA, *β*BA, and AKBA quantification in *Boswellia serrata* extracts. Data are reported as mean ± SD (*n* = 3). Concentration is expressed in mg/g of dry extract (extract A) or mg/ml of hydroenzymatic extract (extract G). For each BA, significant differences between extracts are indicated by ^∗^(*p* < 0.05, Student's *t*-test) and by ^∗∗^(*p* < 0.001, Student's *t*-test).

Extract	KBA^§^	*β*BA	AKBA^§^
A	15.86 ± 0.56^∗∗^	33.53 ± 7.23^∗^	38.30 ± 1.01^∗∗^
G	0.19 ± 0.02	0.50 ± 0.03	0.29 ± 0.04

^§^Data of AKBA concentrations are reported in Beghelli et al. [14].

## Supplementary Materials

Supplementary Materials Table S1: the concentrations of the two boswellic acids analyzed and discussed in the manuscript in five additional dry extracts of *Boswellia serrata*. The table has been added to emphasize the variability of boswellic acid concentration in different dry extracts obtained from the same botanical species.
