# Supplementary material for: Anti-Inflammatory Activity of Boswellia serrata Extracts: An In Vitro Study on Porcine Aortic Endothelial Cells
Source: Oxid Med Cell Longev. 2018 Jun 25;2018:2504305. doi: 10.1155/2018/2504305 (PMC6036794; doi:10.1155/2018/2504305)
Supplement: Supplementary Materials — Table S1: the concentrations of the two boswellic acids analyzed and discussed in the manuscript in five additional dry extracts of Boswellia serrata. The table has been added to emphasize the variability of boswellic acid concentration in different dry extracts obtained from the same botanical species. [file 2504305.f1.pdf]

Supplementary Materials

Table S1. KBA and βBA quantification in five *Boswellia serrata* dry extracts (B-F). Data are reported as mean ± SD (n = 3). Concentration is expressed in mg/g of dry extract.

Sample	KBA Concentration	βBA Concentration
B	28.74±2.73	85.07±5.97
C	nd	nd
D	34.47±3.93	115.35±13.90
E	46.12±6.75	115.56±14.76
F	24.65±1.59	82.70±10.97

nd = not detected

The supplementary material is a table reporting the concentrations of the two boswellic acids analyzed and discussed in the manuscript in five additional dry extracts of *Boswellia serrata*. The table has been added to emphasize the variability of boswellic acid concentration in different dry extracts obtained from the same botanical species.
